# Muscle synergy during free throw shooting in basketball is different between scored and missed shots

**DOI:** 10.3389/fspor.2022.990925

**Published:** 2022-10-06

**Authors:** Naoto Matsunaga, Tomoki Oshikawa

**Affiliations:** ^1^General Education Core Curriculum Division, Seigakuin University, Ageo, Japan; ^2^Faculty of Sport Sciences, Waseda University, Tokyo, Japan

**Keywords:** muscle synergy, non-negative matrix factorization (NMF), success or failure, electromyography (EMG), basketball

## Abstract

The current study investigated the differences in synergy during a free throw in basketball and compared synergies between scored and missed shots. A total of six men's college basketball players participated in this study. A wireless electromyographic system was used to measure the activity of 16 trunk, and upper and lower extremity muscles while completing the free throw. In total, three scored and missed shots each were analyzed to extract the synergies using non-negative matrix factorization. Overall, four synergies were extracted from the successfully made shots, and three synergies were extracted for the missed shot; two synergies were shared between scored and missed shots. The one synergy that contributes to the shoulder flexion was used to set the ball and activate the initial stage of the free throw. Another synergy that contributes the palmar flexion was used to release the ball and activate the final stage of the free throw. The other two synergies in scored shot contribute to lower and upper limb extension in sequence to promote the energy transfer in the middle to the final stage of the free throw. On the other hand, there was only a synergy that corresponded to the middle to the final stage of the free throw extracted from the missed shot. Since the movements of the lower and upper extremity extensions are performed even on a missed shot, we believe that working the from the lower to the upper limb “in sequence,” rather than working the lower and upper limbs “simultaneously,” may influence the success of the shot.

## Introduction

Successful shooting is necessary to win games in basketball; therefore, players devote a significant amount of time to shooting practice. Although several shots, such as dunks and three-pointers, are used in games, players must avoid the defense. However, it is critical to improve the accuracy of free throws because there is no defense involved in such throws, and the distance for the shot is constant. Button et al. (1) reported that players at higher levels of competition have better reproducibility of shooting movements. They develop their shot form through repetitive practice. However, even the most elite athletes can fail, and the factors determining whether a free throw is successful or unsuccessful are unknown. Ball kinematics, such as velocity, release angle, and number of spins, have been reported to influence the success or failure of a free throw (2, 3), and players can control these parameters through repetitive practice. Nakano et al. (4) reported that players have adopted a strategy of minimizing mistakes by slowing down the release of the ball during a free throw. Controlling body behavior is important when considering performance improvements in basketball.

In recent years, there have been studies on muscle synergy while playing sports (5–11). Muscle synergy refers to the concept that functionally similar muscles can be controlled together. The activity of multiple muscles is divided mathematically according to muscle synergy, muscle weighting, and the activation pattern that indicates when muscle synergy occurs. Cheung et al. (5), Matsunaga et al. (6), Matsunaga and Kaneoka (7), Matsuura et al. (9), Sawers et al. (10), and Vaz et al. (11) reported that muscle synergy varies according to the performance level of a sport, such as running, Japanese archery, badminton, swimming, and ballet. Therefore, it is considered that muscle activity and coordination between several muscles influence performance, including that in free throws, because movement is a result of muscle activity. However, it is unclear whether the coordination of different muscles affects the success or failure of free throws. Cheung et al. (5), Matsunaga et al. (6, 8), Sawers et al. (10), and Turpin et al. (12) compared muscle synergies during similar behaviors, such as walking, running, bowing hip extension, and rowing, between experts and beginners. Therefore, we believe that comparisons of synergy are possible even in movements with minor differences between individuals, such as the free throw movement. Thus, the purpose of this study was to clarify whether muscle synergy influences the success or failure of a free throw in basketball. We hypothesized that the same number of synergies would be extracted from both scored and missed shots. Moreover, we expected that muscle synergies would indicate the coordination of different muscles between scored and missed free throws. We also hypothesized that the activation patterns of synergies would vary between scored and missed shots.

## Materials and methods

### Subjects

We recruited six male collegiate basketball players (mean ± SD: age, 20.2 ± 0.8 years; height, 1.74 ± 0.03 m; weight, 63.7 ± 4.5 kg; length of basketball career, 9.7 ± 2.3 years) to participate in this study. All the participants were right-handed. This study was approved by the Ethics Committee of our University (2018-18b). All the subjects read and signed an informed consent form prior to participation in the study.

### Data measurement

After a warm-up, the participants attempted 20 free basketball throws from the free throw line. They shot the ball within 5 s of being handed the ball and did not jump while taking the shot. The participants were not instructed to miss the shot. While the participants were taking a shot, the activity of 16 muscles was recorded using an electromyography (EMG) system (SS-EMGW-HMAG, SPORTS SENSING Co., LTD, Fukuoka, Japan) at 1,000 Hz. The size of the EMG system was 24 mm (W) × 39 mm (D) × 10 mm (H), and it had active electrodes with a distance of 20 mm between the electrodes. Before attaching the EMG system to the participants' skin, the skin was disinfected with alcohol. The EMG systems were attached to the right side of the pectoralis major (PM), deltoid (Del), serratus anterior (SA), latissimus dorsi (LD), triceps brachii (TB), biceps brachii (BB), extensor carpi radialis (ECR), flexor carpi ulnaris (FCU), rectus abdominis (RA), external oblique (EO), internal oblique/transversus abdominis (IO/TrA), erector spinae (ES), gluteus maximus (Gmax), rectus femoris (RF), biceps femoris (BF), and gastrocnemius (GC) muscles. The EMG system attachment position for the PM, Del, SA, TB, BB, ECR, FCU, RA, EO, ES, Gmax, RF, BF, and GC muscles was that used by Perotto et al. (13), and the system position for the IO/TrA muscles was that used by Matsunaga et al. (14). To confirm the start and finish times of the free throw motion, a high-speed camera (LUMIX DC-GH5S, Panasonic Co., Kadoma, Japan) was used. The measuring frequency of the high-speed camera was 200 Hz, and it was synchronized with the EMG system.

### Data analysis

**Figure 1:**
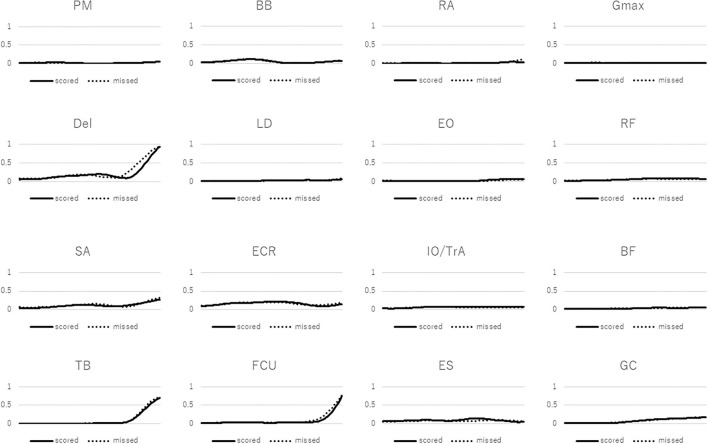
Averaged electromyographic data obtained during free throw shooting. The vertical axis is in arbitrary units. Solid line: scored shot, dotted line: missed shot. PM, pectoralis major; Del, deltoid; SA, serratus anterior; LD, latissimus dorsi; TB, triceps brachii; BB, biceps brachii; ECR, extensor carpi radialis; FCU, flexor carpi ulnaris; RA, rectus abdominis; EO, external oblique; IO/TrA, internal oblique/transversus abdominis; ES, erector spinae; Gmax, gluteus maximus; RF, rectus femoris; BF, biceps femoris; GC, gastrocnemius.

We analyzed three scored and three missed free throws. The data used for the analysis were randomly selected. The start and finish times of a free throw were defined as the “beginning of knee extension movement” and “just after the ball release,” respectively. A custom MATLAB (MATLAB R2020, MathWorks, Inc., Natick, MA, USA) code was used to analyze the EMG data. The raw data were bandpass-filtered between 20 and 450 Hz, and full wave-rectified; the data were standardized using the highest value for each muscle while taking a shot. Thereafter, each data point was interpolated to 201 time points. The average of three shots data for each subject was used as a representative value. Figure 1 depicts averaged data from all free throw shooting sessions. Next, muscle synergies were extracted as follows:


(1) $$\begin{array}{l} E=WC+e \end{array}$$

(2) $$\begin{array}{l} \min_{\begin{array}{c} w>0\\ c>0 \end{array}}{\left||E-WC|\right|}_{FRO} \end{array}$$

where *E* is a *p*-by-*n* initial matrix (*p* is the number of muscles and *n* is the number of time points). The initial matrix comprised normalized EMG data and a cycle for each of the 16 muscles; therefore, *E* is a matrix with 16 rows and 201 columns. *W* is a *p*-by-*s* matrix (*s* is the number of synergies) and represents muscle synergy; C is an *s*-by-*n* matrix and represents the synergy activation pattern; and *e* is a *p*-by-*n* residual error matrix. Formula 2 indicates that matrix “*e*” calculated using formula 1 reaches a minimum. The _FRO_ is Frobenius norm. We used Lee and Seung (15) update rules.

Then, global and local variances accounted for (VAF) were calculated as follows:


(3) $$\begin{array}{l} Global\;VAF=(1-\frac{\sum_{i=1}^{p}\sum_{j=1}^{n}{\left(e_{i,j}\right)}^{2}\;}{\sum_{i=1}^{p}\sum_{j=1}^{n}\;{\left(E_{i,j}\right)}^{2}})\times 100\;[\%] \end{array}$$

(4) $$\begin{array}{l} Local\;VAF\;[m]=(1-\frac{\sum_{j=1}^{n}{\left(e_{m,j}\right)}^{2}\;}{\sum_{j=1}^{n}{\left(E_{m,j}\right)}^{2}\;})\times 100\;[\%] \end{array}$$

where *i* ranges from 1 to *p, j* ranges from 1 to *n*, and *m* represents the muscle. In this study, *i* increases from 1 to 16, and *j* increases from 1 to 201. We selected the least number of synergies that achieved both global VAF > 90% and local VAF > 75%. After the number of synergies was decided, the NMF analysis data for each participant were averaged.

To compare *W* between the scored and missed shots, the scalar product (SP) was calculated as follows:


(5) $$\begin{array}{l} SP=\;\frac{W_{scored}\times W_{missed}}{\left|W_{scored}||W_{missed}\right|}\;\left(0≦SP≦1\right) \end{array}$$

The SP for the use of cosine coefficients can assess the similarity of *W* values between scored and missed shots. We defined the synergy between shots as similar if the SP was above 0.75. These formulas were the same as those used in previous studies (8, 9, 16).

## Results

**Figure 2:**
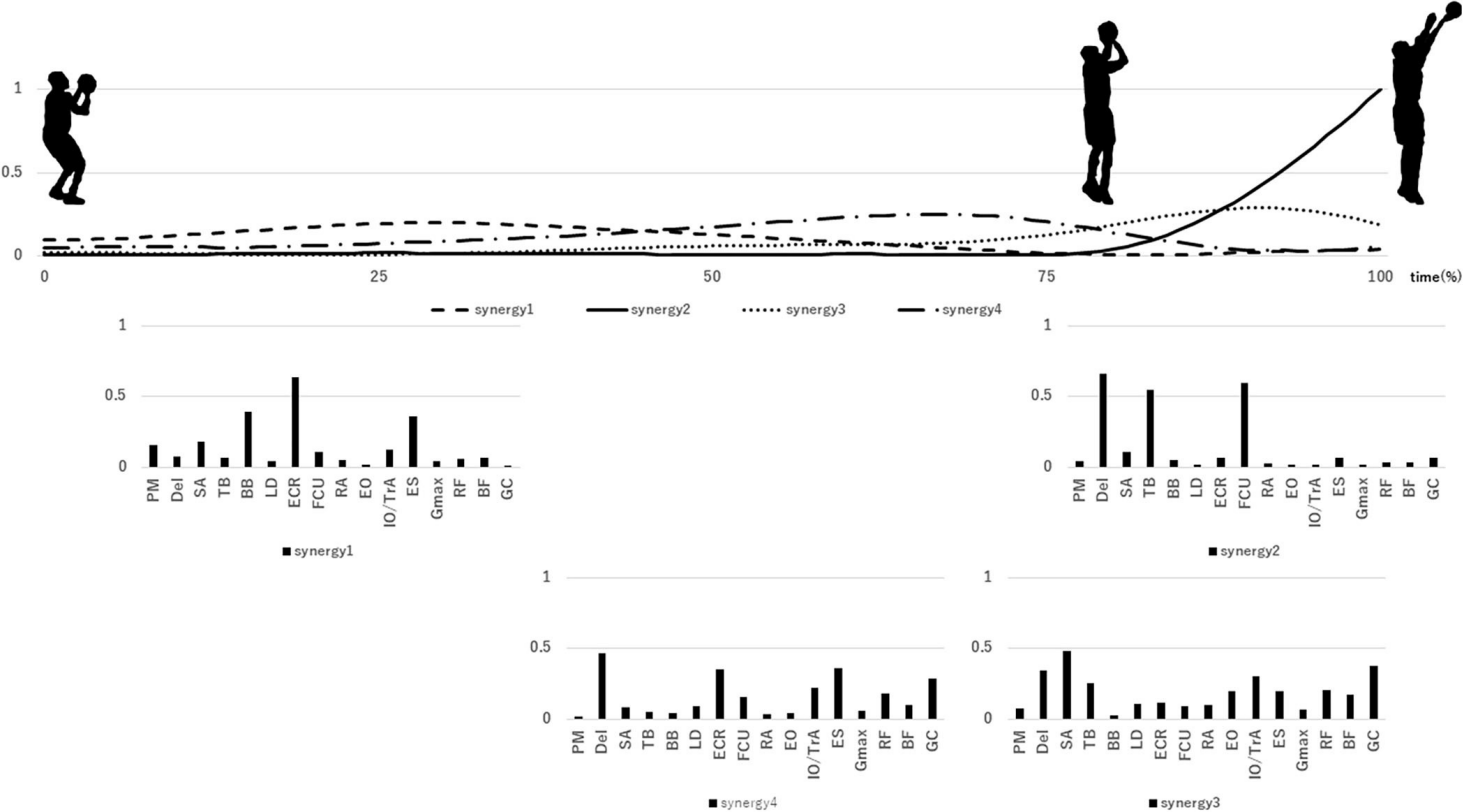
Mean extracted synergies during a scored shot. Upper row: activation pattern of synergies, lower row: extracted synergies. Synergies are posted at the position where the corresponding activation coefficient peaks.

**Figure 3:**
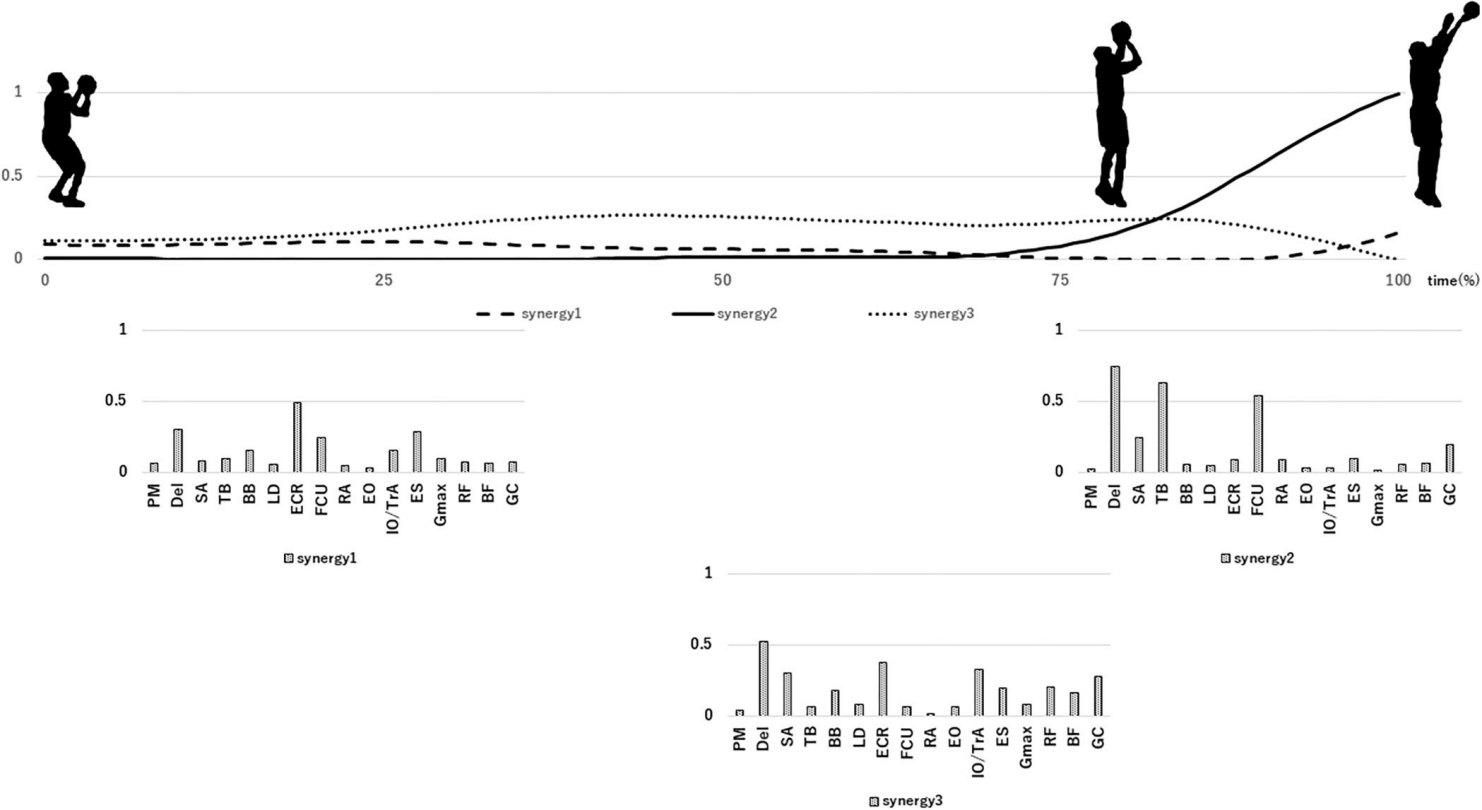
Mean extracted synergies during a missed shot. Upper row: activation pattern of synergies, lower row: extracted synergies. Synergies are posted at the position where the corresponding activation coefficient peaks.

The scored shots were 11.3 ± 2.7 (mean ± SD) out of 20 in this study. Table 1 shows the relationship between global VAF and the number of synergies for both scored and missed shots. If a global VAF did not exceed 90%, then the corresponding number of synergies was rejected (8, 9). The results presented in Table 1 indicate that the number of synergies during the scored shots was three or more. By contrast, there were two or more synergies during the missed shots. Table 2 shows the relationship between local VAF and the number of synergies for scored and missed shots. If a local VAF did not exceed 75%, then the corresponding number of synergies was rejected. The results presented in Table 2 indicate that the number of synergies for scored shots was decided to be four and that for missed shots was decided to be three. Figures 2, 3 reveal the extracted synergy and activation pattern for the scored and missed free throws, respectively. For synergy 1 and synergy 2, the SPs between a scored and a missed shot were 0.88 and 0.98, respectively. Therefore, both the scored and missed shots had these two synergies. The SP between synergy 3 of a missed shot and synergy 3 of a scored shot was 0.87, while the SP between synergy 3 of a missed shot and synergy 4 of a scored shot was 0.93.

**Table 1:** Relationship between the number of synergies and mean global VAF (%).

		**Number of synergies**			
		**1**	**2**	**3**	**4**
Global VAF (%)	Scored shot	81.4 ± 18.7	86.3 ± 16.6	95.4 ± 7.6	98.3 ± 1.6
	Missed shot	87.2 ± 12.1	90.6 ± 11.8	94.6 ± 5.4	98.6 ± 0.7

**Table 2:** Relationship between number of synergies and mean local VAF (%).

		**Number of**	**PM**	**Del**	**SA**	**TB**	**BB**	**LD**	**ECR**	**FCU**	**RA**	**EO**	**IO/TrA**	**ES**	**Gmax**	**RF**	**BF**	**GC**
		**synergies**	
Local VAF (%)	Scored shot	3	74.5	96.1	89.9	96.1	81.7	87.7	91.9	90.2	75.7	84.1	94.5	83.6	73.4	92.2	82.6	89.4
		4	84.1	95.5	95.0	89.2	90.9	91.1	94.1	95.2	86.4	89.0	93.4	84.9	76.4	94.6	85.5	94.1
	Missed shot	2	77.1	96.6	91.3	95.2	80.8	87.9	85.7	91.7	74.1	82.2	83.9	77.8	71.9	89.4	91.7	84.1
		3	78.1	95.5	91.6	95.3	89.7	88.8	93.8	91.6	76.2	81.9	90.7	83.9	87.5	87.8	88.2	81.2

PM, pectoralis major; Del, deltoid; SA, serratus anterior; LD, latissimus dorsi; TB, triceps brachii; BB, biceps brachii; ECR, extensor carpi radialis; FCU, flexor carpi ulnaris; RA, rectus abdominis; EO, external oblique; IO/TrA, internal oblique/transversus abdominis; ES, erector spinae; Gmax, gluteus maximus; RF, rectus femoris; BF, biceps femoris; GC, gastrocnemius.

## Discussion

This study compared the synergies between scored and missed free throws with the main outcome revealing a difference in the number of synergies. These results did not reflect our hypothesis.

Synergy 1 was activated in the initial stage of the free throw. In addition, the Del, SA, ECR, and ES muscles showed high weights in this synergy. These results indicate that synergy 1 promotes a shoulder flexion to set the shooting form. Synergy 2 was activated in the final stage of the free throw. The Del, TB, and FCU muscles showed high weights in this synergy. These results indicate that synergy 2 promotes a palmar flexion to release the ball. Synergy 1 and synergy 2 were shared between scored and missed shots. Therefore, another factor may influence the success or failure of a free throw. Although shooting movement always varies, the variation in movement is smaller during a scored shot than during a missed shot (1, 17). Nakano et al. (4) reported that it is important for the shooting movement of the arm to be constant while taking a shot. Therefore, our findings suggest that synergy 1 and synergy 2 help the upper arm maintain a constant shooting movement.

For synergy 3 of a missed shot, the SP exceeds 0.75 for both synergy 3 and synergy 4 of a scored shot. Synergy 3 of a scored shot was activated in the final stage of the free throw, with the SA, TB, IO/TrA, and GC muscles showing high weights. These findings indicate that synergy 3 mainly promotes upper limb extension before the release of the ball. Synergy 4 of a scored shot was activated in the middle stage of the free throw and corresponded to lower limb extension during the free throw. In case of this synergy, the Del, ES, RF, and GC muscles showed high weights. We believe that synergy 4 promotes lower limb extension while maintaining a shoulder flexion. Rhythmic lower limb extension produces energy to increase ball speed while shooting (18). In addition, the energy to deliver the ball to the basket is transferred from the lower limb and trunk to the upper limb (4). Based on these reports, although the peak activations of synergy 4, synergy 3, and synergy 2 occur in this order, it is thought that energy is first transferred from synergy 4 to synergy 3 and then to synergy 2. Contrarily, synergy 3 of the missed shot was activated from the middle stage to the final stage of the free throw. The activation timing of synergy 3 of a missed shot corresponds to lower limb extension, followed by upper limb extension during a scored shot (synergy 3 and synergy 4 of a scored shot). The Del, SA, ECR, IO/TrA, ES, RF, and GC muscles showed high weights in synergy 3 of a missed shot. Thus, synergy 3 of a missed shot had the characteristics of both synergy 3 and synergy 4 of a scored shot. This might be because both synergy 3 and synergy 4 of scored shot occur simultaneously during a missed shot, rather than in sequence. This result suggests that the missing synergy in case of a missed shot prevents the proper transfer of energy from the lower limb (scored shot synergy 4) to the upper limb (scored shot synergy 3), different from the findings reported by Nakano et al. (4).

There were some limitations to this study. First, this study did not assess or compare the participants' body movements or energy transfer to the ball between scored and missed shots. The variation in shooting movement was less during a scored shot than during a missed shot, although the difference in the variation was not statistically significant (1, 17). The small variation in movement seems to indicate that the force transmission between scored and missed shots is similar. However, body movement or the amount of energy applied to the ball affects the success or failure of a shot. Further research is needed to determine whether synergy affects shooting movement variation or energy transfer to the ball. Second, we analyzed three data sets because the number of failures out of 20 shots was 5 for one participant, and there were three data sets available without noise, such as electrocardiograms, for analysis. Increasing the number of shots may result in more precise data. Third, the number of participants was small. This was because very few players were members of college basketball clubs and had been training continuously in our university. Fourth, this study used global and local VAF for deciding the number of synergies during a shot. The number of synergies in a scored shot was found to be four, and that in a missed shot was found to be three. However, the differences in the VAFs between scored shots and missed shots were not large. Therefore, the difference in the number of synergies may be influenced by the VAF cutoff values. Although we used the same methodology as used in previous studies (7, 19), we believe that cutoff values do need to be considered. Fifth, the success or failure of a shot is influenced not only by body movements but also by biomechanical factors, such as released ball angle and ball speed (20). These factors were not considered in this study. Therefore, it is necessary to take these factors into account in future studies.

## Conclusion

This study investigated muscle synergy during free throws in basketball and compared synergies between scored and missed shots. Our findings revealed a difference in the number of synergies between scored and missed shots; there were four synergies during a scored shot and three synergies during a missed shot. Synergy 3 synergy of a missed shot corresponded to synergy 3 and synergy 4 of a scored shot. Synergy 3 and synergy 4 of a scored shot promoted lower limb and upper limb extension in sequence to transfer energy from the lower limb to the upper limb. Contrarily, it could be that synergy 3 in a missed shot fails to transfer energy from the lower to the upper limb and may influence the success or failure of a free throw. From the perspective of coaching, the results of this study suggest that teaching basketball players to move from the lower to the upper limb in sequence may help them improve their free throw shooting accuracy.

## Data availability statement

The raw data supporting the conclusions of this article will be made available by the authors, without undue reservation.

## Ethics statement

The studies involving human participants were reviewed and approved by Research Ethics Committee of Seigakuin University. The patients/participants provided their written informed consent to participate in this study.

## Author contributions

NM created the main conceptual ideas for the paper. All authors contributed to the manuscript writing.

## Funding

This work was supported by JSPS KAKENHI Grant Number 19K20078.

## Conflict of interest

The authors declare that the research was conducted in the absence of any commercial or financial relationships that could be construed as a potential conflict of interest.

## Publisher's note

All claims expressed in this article are solely those of the authors and do not necessarily represent those of their affiliated organizations, or those of the publisher, the editors and the reviewers. Any product that may be evaluated in this article, or claim that may be made by its manufacturer, is not guaranteed or endorsed by the publisher.
